# Growth and Hope after loss: How TAPS facilitates posttraumatic growth in those grieving military deaths

**DOI:** 10.3389/fpsyg.2022.996041

**Published:** 2022-12-07

**Authors:** Melinda Moore, Jerry K. Palmer, Julie Cerel, Kim Ruocco

**Affiliations:** ^1^Department of Psychology, Eastern Kentucky University, Richmond, KY, United States; ^2^College of Social Work, University of Kentucky, Lexington, KY, United States; ^3^Tragedy Assistance Program for Survivors (TAPS), Washington, DC, United States

**Keywords:** suicide, suicide bereavement, military, military families, peer support, peer mentor

## Abstract

We examined posttraumatic growth for 691 participants of the Tragedy Assistance Program for Survivors (TAPS). Peer mentors of bereaved individuals experienced greater posttraumatic growth (PTG) and reported higher psychological health than those who were non-peer mentors. Active involvement in TAPS and resilience consistently and positively predicted all types of PTG. These prediction models were far stronger (R^2^, AIC) for the suicide-bereaved sample than those bereaved by other causes, and *post-hoc* analyses suggest suicide-bereaved benefitted more than those bereaved by other causes from active participation in TAPS.

## Introduction

Even in times of peace, premature death among active-duty service members remains a constant threat. Each of these deaths is a devastating event for the individuals left behind and may signal increased mental health conditions, such as depression or posttraumatic stress disorder, and place them at risk for complex bereavement disorder (Cozza et al., 2016, 2020). In the broader field of trauma psychology, posttraumatic growth (PTG) or positive changes experienced by survivors of these traumatic events has received recent attention (Tedeschi and Calhoun, 2004), especially among those who are bereaved from military deaths (Moore et al., 2015).

Since 2006, 16,652 active-duty military service members have died while serving in the US armed forces, including 3,863 deaths by suicide. The remaining were combat, accident, illness, and injury-related deaths (Congressional Research Service, 2018). These deaths result in thousands of bereaved. Most military service personnel who die have families. In the Army, Navy, and Air Force, the greatest number of Active-Duty members are married, whereas in the Marine Corps, the greatest number of members have never been married. The Air Force has the highest percentage of married members (54.6%). About two-thirds of Department of Defense (DoD) force dependents are children (62.8%) and about one-third of military dependents are spouses (36.8%; Department of Defense, 2019). Although the study of bereavement in the civilian population is robust, there is little empirical research on the impact of the death of a service member on military families.

Death by suicide remains problematic for all who experience it (Cerel et al., 2014). More than 45,000 Americans die by suicide in the United States every year (Drapeau and McIntosh, 2021) and speculation remains that rates will increase in the aftermath of the COVID-19 global pandemic (Reger et al., 2020). Suicide rates in the US military continue to be the highest since the Defense Department began collecting data in 2001 (Department of Defense, 2019). For every death by suicide, there are 135 people exposed to the death and about 48 individuals who are impacted by that death (Cerel et al., 2017; Cerel et al., 2018). Impact is predicted by perception of closeness to the decedent, not blood kin or first-degree family relationship. Those who perceive themselves as being “close” to the individual who has died by suicide are at higher risk for depression, anxiety, suicidal ideation, and suicide attempt (Cerel et al., 2016; Pitman et al., 2016; Cerel et al., 2018; Maple et al., 2018). Given this new conceptualization of exposure and impact, military “battle buddies” may be significantly impacted by the death of another military member based on their feelings of closeness to the individual who died. Regardless of the cause of death and the potential relationships impacted, the wake of grief left behind in any of these military deaths is significant.

Calhoun and Tedeschi (2006) pioneered the concept of posttraumatic growth (PTG), a construct of positive psychological change that occurs as the result of one’s struggle with a traumatic event. What increases the likelihood of PTG is one’s cognitive engagement with the traumatic event in its aftermath or one’s ability to reflectively engage or “ruminate” over elements of the event in order to repair and restructure one’s understanding of the world. This approach distinguishes between an earlier, involuntary style of rumination, brooding, and a later, voluntary deliberate rumination, reflection. While the first kind of rumination may be associated with early sense-making of an untoward event, the second kind of rumination may be conceptualized as a form of cognitive processing in the aftermath of a crisis that leads to recognition that changes experienced are deeply profound and building of a kind of wisdom. This can manifest itself in several ways. PTG is conceptualized as having five domains or factors within the overall construct, including Relating to Others, New Possibilities, Personal Strength, Spiritual Change, and a deeper Appreciation of Life (Tedeschi and Calhoun, 2004).

Posttraumatic growth (PTG) has been demonstrated to occur for a variety of populations exposed to many different kinds of traumatic events (Nelson, 2011). Military service provides ample opportunity for exposure to traumatic events and, as a result, the occasion to investigate growth within the context of those events (Tedeschi and McNally, 2011). In a study of Vietnam POWs, PTG domains of appreciation of life and personal strength were strongly related to the POWs’ duration of captivity and their own personal characteristic of optimism (Feder et al., 2008). A longitudinal study of a nationally representative sample of military veterans found that 59.4% reported “moderate” PTG in relation to their worst traumatic event. The maintenance or increase of PTG in these veterans relied upon their active engagement in life and development of meaning and purpose, as well as altruism, and gratitude (Tsai et al., 2015).

Peer support models provide an outlet for the development of these constructs of meaning and purpose. They have long been cornerstones of recovery programs for mental illness and addiction, demonstrating significant benefits to those with serious mental health issues over and beyond the benefits of traditional care (Chinman et al., 2014). There is evidence that peer support aids in grief recovery among a broad range of individuals who have experienced sudden and traumatic deaths. To date, there are 32 studies that have investigated the benefits of peer support for bereaved survivors (Bartone et al., 2019), although little is known about why or how successful peer support works. In an examination of best practices of bereavement peer support, several key elements emerged as ingredients of a successful program with particular emphasis on the close matching of peer supporter and the person seeking support in their shared experience of loss (Bartone et al., 2019). Davidson et al. (2012) also found that other benefits of peer support were related to the hope generated through positive self-disclosure and role-modeling, as well as the trust, understanding, and empathy between the peer supporter and the recipient. The term “peer mentor” is also used and tends to describe programs of a longer-term nature, but where there is also a reciprocal effect of the “giving and receiving help founded on key principles of respect, shared responsibility, and mutual agreement of what is helpful” (Mead et al., 2001, p. 135), given the shared “lived experience” of the well-matched peer supporter or peer mentor and recipient.

The elements of what makes for successful peer support or peer mentoring echo some of the basic principles of the facilitation of PTG with an “expert companion” (Tedeschi and Calhoun, 2004). This includes the recognition of the trauma response as a precursor to growth, modeling emotion regulation, constructive self-disclosure, and creation of a coherent trauma narrative with domains of posttraumatic growth (Calhoun and Tedeschi, 2013). If done well, the peer supporter-recipient relationship is one of shared “shattering” of the assumptive worldview and an “existential reevaluation” producing wisdom, life satisfaction, and purpose in life (Calhoun et al., 2010). This matching appears to be critical to the success of a peer support or peer mentor program.

Peer support and peer mentoring programs have been demonstrated to be particularly valuable among suicide-bereaved individuals (Barlow et al., 2010; Bartone et al., 2019). Given the stigma of suicide, suicide peer support may have a counterbalancing effect on the damaging experience of social avoidance or inappropriate comments by those who are confused by the rules for social interaction with the bereaved (Jordan, 2011, p. 287). The grief process of suicide-bereaved has been proposed to be different than that of those bereaved by other types of death (Jordan, 2011). Suicide-bereaved individuals have demonstrated serious health consequences as a result of their exposure to suicide and impact of such a death, as previously articulated. They are likely to feel isolated and stigmatized (Mitchell et al., 2003). They may experience an intense “shattering” of their assumptive worldview (Janoff-Bulman, 2006), an opportunity for reconstruction of their life narrative (Neimeyer, 2006), and greater struggle cognitively with the death and rumination on the “meaningfulness” of the traumatic event (Calhoun and Tedeschi, 2006). However, one longitudinal study of suicide-bereaved individuals (Levi-Belz, 2019) found that self-disclosure and social support play important roles in facilitating PTG among these unique trauma survivors. While the experiences of suicide-bereaved are traumatizing and distressing, peer support or mentoring among suicide-bereaved may provide ample opportunity to produce PTG.

Few studies have examined PTG among bereaved military families or explored the impact of peer support or peer mentoring on the suicide-bereaved and the reciprocal effect on the peer supporter or mentor The question is not does growth exist, but how do we facilitate it among trauma survivors? The present study proposes to examine one model program’s ability to facilitate PTG among its recipients and to also examine the effects of their peer mentor program on both the peer mentor and the participant of the program.

The Tragedy Assistance Program for Survivors (TAPS) is a national nonprofit 501c3 organization that was formed in 1994 to provide ongoing emotional help to all who are grieving the death of a loved one from all causes of death in military service. Over 90,000 bereaved adults and children have been helped by TAPS since its inception. This includes 12,000 suicide-bereaved military family members. The rest of those served included bereaved from other sudden and traumatic causes (SAT) causes of death, including combat and training accidents.

Every year, 10,000 adults and 3,640 children participate in programs, retreats, and regional seminars geared toward individuals who have lost a military loved one. TAPS conducts twice-monthly regional events, as well as retreats tailored to grieving parents, children, and spouses of these fallen war heroes. TAPS Regional Seminars feature activities, workshops, small group discussions, memorial celebrations, and special events. The signature National Military Survivor Seminar and Good Grief Camp held over Memorial Day Weekend has been conducted for 26 years to provide a weekend of understanding, hope, and courage within the context of the nation’s Capital and the beautiful monuments that highlight their loved one’s service. Since 2009, as suicide rates in the US Armed Forces dramatically increased (Reimann and Mazuchowski, 2018), TAPS has created specific programming for those grieving the suicide loss of a military member. Suicide-bereaved individuals represent a significant portion of those who are served with about 1,000 suicide-bereaved individuals attending the suicide-specific event annually and represent about 30% of all TAPS programs. The National Military Suicide Survivor Seminar and Good Grief Camp for Young Survivors were specifically designed to meet the unique needs of the suicide-bereaved. The TAPS Suicide Postvention Model (Ruocco et al., 2021) was articulated and described in October 2019 at the National Military Suicide Survivor Seminar. The TAPS Suicide Postvention Model follows the TAPS’ peer-based model of care, integrating suicide-specific programing around emotional distress, as well as best practices in grief and trauma. By connecting programming, peers, services, and resources that specifically addressed their needs in an organized way, suicide-bereaved are able to appreciate the changes in them that can be identified as posttraumatic growth.

The TAPS Suicide Postvention Model dovetails with the PTG Facilitation Program proposed by Tedeschi and McNally (2011). The PTG Facilitation Program includes five distinct phases: psychoeducation, management of distress, constructive self-disclosure, coherent life narrative development, and articulation of revised or new principles by which to live their life, buffer against future events, and provide meaning and purpose (Tedeschi and McNally, 2011, p. 147–148). TAPS provides “expert companionship” throughout their programming, which is a hallmark of a successful PTG Facilitation Program (Tedeschi and McNally, 2011, p. 149). Expert companions, in the form of peer mentors, are critical to this process and may provide the suicide-bereaved and those bereaved by other causes with the ability to translate these new principles into their everyday lives, helping them to take an active role in this process, integrate them into their new identity, including new possibilities and pathways, providing meaning and purpose, for their life.

The heart of TAPS’ service is the peer mentor network. TAPS provides long-term, peer-based emotional support, crisis response and intervention, casualty casework assistance, and grief and trauma resources and information. There are over 30 individual contacts made with bereaved participants by peer mentor specialists each year, including but not limited to telephone conversations, remembering birthdays with cards, and providing information and magazines. Every day, there are 17 new bereaved participants who join TAPS and then rolled into the support of the network of peer mentor specialists. These peer mentor specialists were once TAPS participants but have at least 2 years from their bereavement experience and have received specialized training.

Our hypotheses were:

1. Peer mentors will report higher posttraumatic growth.
2. Peer mentors will report better psychological health.
3. A meaningful life positively predicts posttraumatic growth. We operationalized a meaningful life as actively participating in an organization (TAPS) designed to help other-bereaved persons and specifically hypothesize that active involvement in TAPS predicts posttraumatic growth.
4. This active involvement in TAPS will also predict better psychological health.
5. Suicide-bereaved respondents will experience greater posttraumatic growth.
6. Consistent with much prior research, resilience will positively predict posttraumatic growth.

## Materials and methods

### Participants

From March 2017 to August 2017, recipients of TAPS services received an invitation through email to participate in the research and to complete the survey questions online *via* Survey Monkey. This included both suicide-bereaved and non-suicide-bereaved participants in TAPS. Other participants were contacted directly at TAPS’ National Military Survivor Seminar and Good Grief Camp for Young Survivors during Memorial Day Weekend 2017.

Participants could either reply to survey questions *via* a link in the email or *via* computers set up at booths during the 2017 Memorial Day conference. The survey included questions measuring posttraumatic growth, resilience, and several measures of psychological well-being, such as posttraumatic stress disorder, depression, and anxiety.

Six hundred ninety-one participated. The average age of the survey respondent was 52 years 9 months (SD = 11.8, range 20 to 86). Four hundred eighty-one (85%) were female, 83 (15%) were male, one was non-binary, and 126 did not answer this question. Four hundred sixty-seven (66.1%) identified as Caucasian/white. Thirty-six (5.2%) identified as African American, 29 (4.2%) as Hispanic, 15 (2.2%) as Native American, five as Asian, and four as multi-race.

### Peer mentors

Ninety-five (21%) identified as peer mentors and 365 (79%) indicated they were not. Most peer mentors had served as a peer mentor for about three years, with the average being 3.4 years and the range being from brand new to 20 years. About one-fourth of these reported not having mentored anyone, though these were mostly newly trained peer mentors. Of those who had mentored, most (87%) had mentored between one and seven. One respondent reported having mentored “about 70” over two decades and another reported having mentored “about 25.”

### Recipient of peer mentor services

Two hundred forty-two (43%) indicated they had received peer mentor services, while 315 (57%) indicated they had not. One hundred sixty-one (39%) indicated they had received help from “unofficial mentors” while 256 (61%) said they had not.

### Years since loved one’s death

On average, 7 years had transpired since the death of the respondent’s loved one. The range was from 49 years to less than 1 year, although 95% of the deaths had occurred within the previous 15 years.

### Closeness to the decedent

Eighty-seven percent indicated they were “very close” to the decedent and 94% indicated they were either “close” or “very close” to the decedent.

### Effect of the death

Eighty-one percent indicated that the death “had a significant or devastating effect on me that I still feel.” A further 15.6% indicated that the death “disrupted my life in a significant or devastating way, but I no longer feel that way.”

### Cause of the death

One hundred eighty-two (41%) indicated that the loved one died by suicide, 94 (21%) in combat, 81 (18%) of “other” causes, 66 (15%) in an accident, and 17 (4%) of natural causes.

### Active in TAPS

Two hundred fifty-four (46.2%) indicated they were minimally involved in TAPS, 138 (25.1%) were moderately involved. One hundred and twenty-eight (23.3%) were “not at all” involved, and 30 (4.3%) were highly involved. Related to this, 389 of 431 (90%) said they did not provide “other services” to TAPS.

### Closeness of TAPS relationships

One question asked respondents “How many TAPS individuals, including peer mentors, mentees, etc., have you become close to?” The question was open-ended, making estimation of an exact average difficult, but based on the pattern of responses it appears the average number is about five.

### Satisfaction with TAPS services

Seventy percent (380 of 544) of respondents indicated they were “highly satisfied” with TAPS services, 18 percent (99) indicated they were “satisfied,” 8 % (43) were “somewhat satisfied,” and 4 % (22) were not satisfied.

Related to this, 64 percent (335 of 521) answered that there were things they could do now that they were not able to do before becoming involved with TAPS. Another question related to this was “Is there anything you were not able to receive help with?” Sixty-seven percent (273 of 408) said “no” and the “yes” answers varied considerably, including things not under TAPS control. These answers are presented later in this document. Forty percent (158 of 392 responding) found TAPS Magazine “extremely helpful,” 32.7% found the magazine very helpful, 22% found it somewhat helpful; 5% did not find it helpful.

## Measures

Posttraumatic Growth was measured using the Posttraumatic Growth Inventory (PTGI; Tedeschi and Calhoun, 1996), a 21-item instrument for assessing positive outcomes in people who have experienced traumatic events. There is an overall PTG score and five domain scores culled from five subscales: personal strength, relating to others, new possibilities, appreciation for life, and spiritual growth. The items of the scale are a series of positively worded statements and participants are asked to use the statements to indicate the degree to which change occurred in their life as a result of their crisis. Participants respond to each of the 21 items on a six-point Likert-type scale, ranging from 0 (*I did not experience this change as a result of my experience*) to 5 (*I experienced this change to a very great degree*). Posttraumatic Depreciation was assessed *via* the 21-item measure (PTD-21) developed by Baker et al., 2008. This measure consists of negatively worded items otherwise identical to those of the PTG-21, and assesses of the opposite of growth, depreciation. The five factors of the PTD-21 are likewise identical to those of the PTG-21.

The Resilience Scale (RS-14; Wagnild, 2009) is a shortened, authorized version of the 25-item Resilience Scale (Wagnild and Young, 1993) that assesses adults’ trait of resilience on a 7-point Likert-type scale. The internal consistency of the RS-14 has been found to range from 0.76 to 0.91. Test–retest reliability has been reported range between 0.67 and 0.84.

Participation in TAPS was measured by one four-point item, “How active are you in TAPS?” Endpoints ranged from zero (*Not at all*) to four (*Highly active*).

Posttraumatic Stress Disorder Checklist. The Posttraumatic Stress Disorder Checklist (PCL; Weathers et al., 1993) is a 17-item measure designed to assess the symptoms of PTSD as described in the Diagnostic and Statistical Manual of Mental Disorders (4th ed. [DSM–IV]; American Psychiatric Association, 2000). The PCL uses a 5-point Likert-type response format with options ranging from 1 (*not at all*) to 5 (*extremely*) and scores on the PCL range from 17 to 85, with higher scores indicating more endorsed symptoms of PTSD symptomology.

The exposure and impact of suicide were measured by the Suicide Exposure Experience Screener (SEES; Maple et al., 2022) is a brief screener assessing experience of exposure to suicide with psychological distress. The screener has two items: one item to assess participants’ reported closeness to the person who died by suicide and the second item to assess participants’ reported impact of this death on them. Closeness is assessed on a 5-point Likert scale ranging from 1 (*Not close*) to 5 (*Very close*) in response to the question: “How close would you describe your relationship with the person who died?” Impact is assessed on a 5-point Likert scale ranging from 1 (*Had little effect on my life*) to 5 (*Had significant/devastating effect on me that I still feel*) in response to the question: “What effect did this death have on your life?” It has been demonstrated to have high reliability (*r_sp_* = 0.72 to.83) and concurrent validity.

Suicidality was measured using the Suicide Behaviors Questionnaire Revised (SBQ-R; Osman et al., 2001), a 4-item self-report instrument that taps four dimensions of suicidality (coefficient alphas 0.76–0.88).

Depression was measured by the Patient Health Questionnaire (PHQ-9), a multipurpose instrument for “screening, diagnosing, monitoring, and measuring the severity of depression” (Kroenke et al., 2001). The diagnostic validity of the 9-item PHQ-9 was established in studies involving 8 primary care and 7 obstetrical clinics. PHQ-9 scores >10 had a sensitivity of 88% and a specificity of 88% for Major Depressive Disorder. Reliability and validity of the tool have indicated it has sound psychometric properties. Internal consistency of the PHQ-9 has been shown to be high. A study involving two different patient populations produced Cronbach alphas of 0.86 and 0.89 (Kroenke et al., 2001).

Anxiety was measured using the Spitzer et al. (2006) measure of Generalized Anxiety Disorder (GAD-7). The GAD-7 represents an anxiety measure based on seven items which are scored from zero to three. The whole scale score can range from 0 to 21 and cutoff scores for mild, moderate, and severe anxiety symptoms are 5, 10, and 15, respectively. At the cutoff score of 10 both sensitivity as well as specificity exceed 0.8, so that the operating characteristic of the scale, based on using a structured psychiatric interview as the criterion, is satisfactory. Internal consistency of the GAD-7 was estimated at 0.92 and convergent validity was established by means of correlations with two other anxiety measures. I.

## Results

Reliabilities for the PTG dependent variables ranged from 0.71 (appreciation for life) to 0.93 (overall PTG) and ranged from 0.93 (resilience) to 0.96 (PTSD) for the predictors (See Table 1). Descriptive statistics and intercorrelations are presented in Table 1.

**Table 1:** Descriptive statistics and correlations.

	1	2	3	4	5	6	7	8	9	10	11	12	Mean	SD
1. PTG	0.93												2.57	1.13
2. PTG-R	0.87^*^	0.86											2.37	1.27
3. PTG-N	0.85^***^	0.62^***^	0.81										2.40	1.33
4. PTG-P	0.84^***^	0.60^***^	0.70^***^	0.81									2.69	1.40
5. PTG-A	0.82^***^	0.61^***^	0.66^***^	0.71^*^	0.71								3.13	1.35
6. PTG-S	0.63^***^	0.48^***^	0.42^***^	0.44^***^	0.43	0.79							2.53	1.87
7. Res	0.36^***^	0.27^***^	0.35^***^	0.37^***^	0.29^***^	0.13^**^	0.93						5.36	1.10
8. Active	0.29^***^	0.25^***^	0.33^***^	0.25^***^	0.20^***^	0.08	0.18^***^	−					1.15	0.83
9. Years	0.09^*^	0.01	0.13^**^	0.14^**^	0.07	0.05	0.10^*^	0.02	−				6.70	5.28
10. PTSD	−0.18^***^	−0.20^***^	−0.17^***^	−0.17^***^	−0.13^*^	−0.02	−0.65^***^	−0.13^**^	0.11^*^	0.96	−		2.17	1.01
11. Age	−0.02	0.00	−0.05	−0.09	0.05	0.12^*^	0.06	−0.10^*^	0.09	0.20^***^			53.67	11.59
12. Close	0.03	0.05	0.00	0.09	−0.11^*^	0.01	−0.02	−0.03	0.04	0.07	0.03	−	4.80	0.63

Coefficient Alpha in main diagonal; PTG-R, Relating to Others; PTG-N, New Possibilities; PTG-P, Personal Strength; PTG-A, Appreciate Life; PTG-S, Spiritual; Res, Resilience; Active, Active in TAPS; Years, Years since the death; PTSD, Posttraumatic Stress; Close, Closeness to Decedent.

* *p* < 0.05;

** *p* < 0.01;

*** *p* < 0.001.

### Effects of peer mentorship on growth and health

We used MANOVA to test the effects of peer mentorship on PTG (Hypothesis one). The multivariate test for the five PTG subscales was significant for both peer mentorship (Wilks Λ = 0.930, *p* < 0.001) and cause of death (Wilks Λ = 0.941, *p* < 0.001). Follow-up univariate ANOVAs for each of the PTG dimensions produced significant main effects of peer mentorship and new possibilities [*F*(1,378) = 14.4, *p* < 0.001, d = 0.50], peer mentorship and personal strength [F(1,378) = 15.1, *p* < 0.01, d = 0.34], and cause of death and spiritual [F(1,378) = 4.05, *p* < 0.05, d = 0.21]. For these, being a peer mentor resulted in higher growth and those whose loved ones died by suicide grew more spiritually. No interactions were significant.

For hypothesis two, we computed separate 2 × 2 ANOVAs on PTSD, PHQ, GAD, and SBQ by peer mentorship and cause of death. Peer mentorship was significant for PTSD [*F*(1, 330) = 9.5, *p* < 0.01, d = 0.48], PHQ [*F*(1, 333) = 4.26, *p* < 0.05, d = 0.34], GAD [*F*(1, 338) = 5.74, *p* < 0.01, d = 0.40], and for SBQ [*F*(1, 325) = 4.46, *p* < 0.05, d = 0.33]. For these, peer mentorship resulted in better health outcomes (e.g., less suicidal, less anxious). The cause of death was not significant, and no interactions were significant.

### Predicting growth

To test hypotheses three, four, and six, we regressed overall PTG and each of the PTG domains onto resilience and participation in TAPS. Also included as variables of interest were PTSD, years since the death, respondent age, and level of closeness with the decedent. These regression results were done separately for suicide-bereaved and other-bereaved and are presented in Table 2.

**Table 2:** Regressions.

Dependent	Predictor	β		r_**y**_**(x-x)**	
		Died by Suicide	Died by Other Causes	Died by Suicide	Died by Other Causes
Overall PTG ${\hat{R}}_{1}^{2}=0.304$ ${\hat{R}}_{2}^{2}=-0.096$ AIC_1_ = −3.2 AIC_1_ = 37.3	Resilience	**0.526** ^***^	**0.292** ^***^	0.368	0.215
	Active in TAPS	**0.264** ^***^	**0.155** ^*^	0.251	0.150
	PTSD	**0.278** ^**^	0.051	0.189	0.036
	Years Since Death	0.135	0.047	0.130	0.046
	Closeness	0.093	0.023	0.088	0.022
	Age	−0.070	0.034	−0.066	0.032
Relate to Others ${\hat{R}}_{1}^{2}=0.179$ ${\hat{R}}_{2}^{2}=0.33$ AIC_1_ = 47.1 AIC_1_ = 88.2	Resilience	**0.355** ^***^	0.152	0.249	0.112
	Active in TAPS	**0.271** ^***^	0.129	0.258	0.125
	PTSD	0.069	0.009	0.047	0.007
	Years Since Death	0.043	−0.040	0.041	−0.039
	Closeness	0.028	−0.014	0.026	−0.013
	Age	−0.042	0.095	−0.040	0.090
New Possibilities ${\hat{R}}_{1}^{2}=2.77$ ${\hat{R}}_{2}^{2}=0.159$ AIC_1_ = 40.1 AIC_1_ = 99.6	Resilience	**0.523** ^***^	**0.331** ^***^	0.367	0.243
	Active in TAPS	**0.285** ^***^	**0.250** ^***^	0.272	0.242
	PTSD	**0.282** ^**^	0.090	0.191	0.064
	Years Since Death	0.144	0.098	0.138	0.096
	Closeness	0.071	0.004	0.068	0.004
	Age	−0.023	−0.008	−0.022	−0.008
Personal Strength ${\hat{R}}_{1}^{2}=0.367$ ${\hat{R}}_{2}^{2}=0.100$ AIC_1_ = 60.8 AIC_1_ = 123	Resilience	**0.631** ^***^	**0.311** ^***^	0.442	0.229
	Active in TAPS	**0.156** ^*^	0.127	0.148	0.123
	PTSD	**0.318** ^**^	0.019	0.216	0.014
	Years Since Death	**0.198** ^**^	0.038	0.190	0.037
	Closeness	**0.156** ^*^	0.081	0.148	0.079
	Age	−0.113	−0.063	−0.107	−0.059
Appreciate Life ${\hat{R}}_{1}^{2}=0.216$ ${\hat{R}}_{2}^{2}=0.066$ AIC_1_ = 74.5 AIC_1_ = 120	Resilience	**0.305** ^**^	**0.284** ^**^	0.214	0.209
	Active in TAPS	**0.192** ^*^	0.097	0.183	0.094
	PTSD	**0.222** ^*^	0.083	0.151	0.058
	Years Since Death	**0.188** ^*^	0.050	0.180	0.048
	Closeness	0.005	0.042	0.005	0.040
	Age	−0.157^*^	−0.065	−0.149	−0.061
Spiritual ${\hat{R}}_{1}^{2}=0.48$ ${\hat{R}}_{2}^{2}=0.56$ AIC_1_ = 190 AIC_2_ = 263	Resilience	**0.254** ^*^	0.167	0.178	0.123
	Active in TAPS	0.089	−0.045	0.084	−0.043
	PTSD	**0.363** ^**^	0.020	0.246	0.014
	Years Since Death	−0.004	0.103	−0.003	0.125
	Closeness	0.132	0.003	0.125	0.002
	Age	0.094	**0.183** ^*^	0.089	0.173

${\hat{R}}_{1}^{2}=\mathrm{Adjusted}\;R^{2}\;\mathrm{Suicide}$ ; ${\hat{R}}_{2}^{2}=\mathrm{Adjusted}\;R^{2}\;\mathrm{Other Causes}$ ; AIC_1_, Akaike Information Suicide; AIC_2_, Akaike Information Criterion Other Causes df, 7,135 for Died by Suicide Group; 7,196 for Died by Other Causes Group.

Bold values indicates that these are statistically significant.

* *p* < 0.05;

** *p* < 0.01;

*** *p* < 0.001.

### Suicide-bereaved

Resilience positively and significantly predicted PTG overall and for all PTG domains. In fact, the beta weights for resilience were consistently stronger than those of any other. Being active in TAPS significantly predicted growth for overall PTG and for four of five domains, though not for spiritual growth. Posttraumatic stress symptoms also significantly predicted growth for four of five domains, though not for relating to others. Years since the death and closeness to the decedent positively predicted personal strength and years since the death positively predicted appreciation for life, while suicidality, and age negatively predicted appreciation for life.

Just over 30 percent of the variance in overall growth was accounted for by the predictors. Between 5% (spiritual) and 37% (personal strength) of the domain variances were accounted for.

### Other-bereaved

Both resilience and being active in TAPS predicted overall PTG for other-bereaved and the new possibilities domain. However, only resilience predicted for personal strength and appreciation for life and none predicted relating to others. Age positively predicted the spiritual growth domain.

About 10% of overall PTG was accounted for by the predictors, and for domains, this ranged from about 3% (relating to others) to 28% (new possibilities).

### Suicide vs. other-bereaved

To test hypothesis five, we computed *t*-tests comparing suicide- and other-bereaved on the PTG variables. The overall PTG difference was non-significant (*p* > 0.05), but relating to others [t(372) = 1.73, *p* < 0.05, d = 0.18] and spiritual [t(372) = 1.88, *p* < 0.05, d = 0.20] were in the expected direction, with growth being higher for the suicide-bereaved group.

To further investigate possible reasons for differences between the suicide- and other-bereaved regression models, we computed *post-hoc t*-tests of the following additional items: “Were you seeking help when TAPS contacted you?” (*1 = yes/0 = no*), “how satisfied are you with TAPS services?” (*1 = not at all, 4 = highly*), and “Are there things you are able to do now that you were not before you became involved with TAPS?” (*1 = yes/0 = no*). After Bonferroni adjustment all three were significant. Those in the suicide-bereaved group were more likely to have been seeking help [t(367) = 2.65, *p* < 0.05, d = 0.28], more satisfied with TAPS’ services [t(363) = 3.17, *p* < 0.01, d = 0.35], and were more likely to report that there were things they could do now that they could not before [t(356) = 2.6, *p* < 0.05, d = 0.28].

## Discussion

Results overall support the hypotheses. Active involvement in the TAPS organization and an individual’s resilience positively and significantly predicted overall PTG for both the suicide-bereaved and for those bereaved by other causes. For the suicide-bereaved, active involvement and resilience predicted four of five specific PTG domains. For those TAPS participants who were bereaved by other causes, being active in TAPS predicted the domain of new possibilities, whereas resilience predicted four of five domains. The TAPS Suicide Postvention Model for the suicide-bereaved provides structured events and unstructured support that affords its participants with the elements necessary for meaning reconstruction of their specific and traumatic loss. This supports assertions made by Neimeyer and other social constructionist theorists (Neimeyer, 2006; Neimeyer et al., 2014) that PTG is possible when done so in an environment of safety and the loss survivor is able to “construct” their understanding of the traumatic event within the context of expert companionship or peer mentors. What seems related to the likelihood of PTG is one’s mental engagement with the loved one’s death and one’s ability to reflectively engage or “ruminate” over elements of the event in order to repair and restructure their understanding of the event and their own reconstructed world within the context of a shattered “assumptive world” view (Janoff-Bulman, 2006).

The hypothesis that suicide-bereaved participants will experience greater posttraumatic growth received some support. PTG domains of relating to others and spiritual were higher for the suicide-bereaved group. The most noticeable difference between the suicide-bereaved and other-bereaved groups was the differences in growth reflected by the prediction models. The regression models were far stronger for the suicide-bereaved group relative to the other-bereaved group. More variance was accounted for in the dependent variables and more predictors were significant. Confirming the model differences, the Akaike information criteria for suicide-bereaved regression models were consistently and strongly better (lower values) than those for the other-bereaved group (See Table 2). Regression models for suicide-bereaved fit better. This occurred despite the larger sample size for the other-bereaved group and may be explained by this study’s finding of significant differences between those who are exposed to a suicide death and those who are not exposed to a suicide death.

Reconstruction of one’s understanding of their core beliefs about themself and their life, their “assumptive world,” may be especially true of the suicide-bereaved. Independent of groups such as TAPS, suicide-bereaved individuals may experience levels of social stigma and isolation (Mitchell et al., 2003), making it difficult for them to safely engage the necessary ruminative processes combined with expert companionship to produce PTG. Lowering of distress and feeling connected and cared for may assist in one’s ability to effectively ruminate on the traumatic event, leading to recognition that changes experienced within them are deeply profound and building of a kind of wisdom. While TAPS’ programming may be effective at addressing this need for social support, providing the necessary psychoeducation to manage distress and help the bereaved approach the necessary work of ruminating the trauma, expert companions or peer mentors may enhance and greatly facilitate the process.

The hypothesis that all peer mentors, both suicide-bereaved and non-suicide-bereaved, scored better across all mental health indicators, was supported. These outcomes are emblematic of the phenomenon inherent in the benefit of giving back to one’s community. There is something valuable to the health and well-being of the peer mentor by volunteering their time to help those TAPS recipients who are newly bereaved. They are finding purpose and meaning, but they are also tangibly seeing results in their health indicators. This does not imply that the peer mentors are without distress. Suicidality, PTSD symptoms, depression, and anxiety are all present, but, overall, their well-being is better than that of the others. The concept of “altruism born of suffering” (ABS, Staub and Vollhardt, 2006) is tangibly demonstrated by the TAPS peer mentor program. These are individuals who have been TAPS participants and recipients of services previously and are now generously giving back to help those bereaved individuals who are now standing in the very “shoes” of their own traumatic walk. As Holocaust survivor and psychiatrist Viktor Frankl (2006) describes, “life is potentially meaningful under any conditions, even those which are most miserable” (p. 137). According to Frankl, who wrote *Man’s Search for Meaning* in the aftermath of his experiences at the Nazi concentration camps, it was the uncertainty surrounding how long prisoners would be in camps that were the most depressing part. Because of this, prisoners were not able to aim for the ultimate goal in life and ceased living for the future. This doomed them, just as it dooms those who survive traumas, such as losing a loved one to suicide. Living by looking toward the future guards against decay, as does finding meaning and purpose in our lives. TAPS peer mentor program may provide both meaning and purpose to those who are the peer mentors, as well as a future-oriented perspective that preserves their own PTG’s longevity.

TAPS services appear to provide a sense of connection and belongingness for military families, meaning in their loss, and a new sense of purpose. The TAPS Suicide Postvention Model represents what is supported in the research literature about programs that facilitate PTG. These programs provide psychoeducation, management of emotional distress, constructive self-disclosure, and development of coherent narratives, leading to articulation of new life narratives and done within the context of expert companionship utilizing peer support (Tedeschi et al., 2018).

Knowing that growth after traumatic loss is possible provides hope to the individual who has experienced the loss, but also gives them tools for rebuilding their lives by giving them a real understanding of how they have been changed as a result of this trauma. Facilitating posttraumatic growth is becoming an important therapeutic approach that both professionals and organizations serving those who have experienced traumatic loss may employ (Tedeschi and McNally, 2011; Calhoun and Tedeschi, 2013). As TAPS appears to foster this kind of growth in bereaved military families, it provides a perfect venue to learn how some families who have been through the worst are able to come out of this traumatic life experience in a way that their lives are forever changed, but more resilient and robust than they might have otherwise been.

Admittedly, our measure of active involvement in TAPS was broad. TAPS participants can be involved in the organization in many ways, including formally mentoring others who are also bereaved, informally supporting other members, and volunteering for a wide variety of positions and events at the biannual conference, among others. Future research should examine the various ways one can become involved and attain meaning for their lives and whether some types result in more growth than others.

Although the regression model suggested PTSD positively predicts PTG, the zero-order correlations suggest a positive relationship. Clearly, the regression results were influenced by the multicollinearity between PTSD and other predictors. In other words, after resilience and being active in TAPS were entered into the equation, the remaining shared variance between PTSD and PTG was positive. Whereas some amount of trauma must occur for any growth to take place, it is entirely possible that amount or type of trauma may moderate this. Future research should investigate the relationship between PTSD symptoms and PTG. Some have suggested the relationship to be negative (Butler et al., 2005) and others have suggested a linear relationship (Hall et al., 2010), and others have a more complex curvilinear relationship (Dekel et al., 2012). In this study, growth was experienced within the context of trauma. They are not separate dimensions, but co-occurring, especially as a certain amount of trauma is necessary for growth to occur. The shattering of the “assumptive world view,” the traumatic symptoms created, and the cognitive dissonance created by this traumatic experience actually lays the groundwork for the possibility of growth.

Suicide-bereaved may find a profound sense of belonging, being understood, and freedom to approach their painful emotions through this experience of participation in TAPS. It is the safety of TAPS, the lowering of the distress associated with being with peers, which allows for the internalization of the programming and other important learning that occurs in this environment. Bereaved may begin to privately reflectively ruminate, a critical step in the facilitation of PTG, and appreciate the changes that have occurred within them as the result of their traumatic experience. The context of support, expertise, and validation of peer mentors and activities that help them explore this new interpretation of their traumatic experience gives them space to create new narratives, new goals, new hopes, and aspirations. These are not illusory or “finding” of benefit, but profound changes that signal growth. The main limitation of the study is that it is based on self-report, which could introduce some response bias. It is not clear how a larger nationally representative sample might yield different results. This is a very unique sample, military suicide-bereaved and military bereaved by other causes. The pattern of findings for different samples might not be the same.

## Data availability statement

The raw data supporting the conclusions of this article will be made available by the authors, without undue reservation.

## Ethics statement

The studies involving human participants were reviewed and approved by Eastern Kentucky University Institutional Research Board. The patients/participants provided their written informed consent to participate in this study.

## Author contributions

MM and JP provided substantial contributions to the conception and design of the work and the acquisition, analysis, and interpretation of data, as well as drafting the work. JC and KR provided substantial contributions to the conception, design, and execution of the work. All authors contributed to the article and approved the submitted version.

## Funding

The research conducted was supported by funds from the Tragedy Assistance Program for Survivors (TAPS).

## Conflict of interest

The authors declare that the research was conducted in the absence of any commercial or financial relationships that could be construed as a potential conflict of interest.

## Publisher’s note

All claims expressed in this article are solely those of the authors and do not necessarily represent those of their affiliated organizations, or those of the publisher, the editors and the reviewers. Any product that may be evaluated in this article, or claim that may be made by its manufacturer, is not guaranteed or endorsed by the publisher.
